# Safety and outcomes of percutaneous tracheostomy in coronavirus disease 2019 pneumonitis patients requiring prolonged mechanical ventilation

**DOI:** 10.1017/S0022215120002303

**Published:** 2020-11-04

**Authors:** A Takhar, C Tornari, N Amin, D Wyncoll, S Tricklebank, A Arora, I Ahmad, R Simo, P Surda

**Affiliations:** 1 Department of Otolaryngology and Head and Neck Surgery, Guy's and St Thomas’ NHS Foundation Trust, King's College London, London, UK; 2 Department of Critical Care, Guy's and St Thomas’ NHS Foundation Trust, King's College London, London, UK; 3 Department of Anaesthesia, Guy's and St Thomas’ NHS Foundation Trust, King's College London, London, UK

**Keywords:** SARS-CoV-2, Coronavirus, Tracheostomy, Mechanical Ventilation, Airway Management

## Abstract

**Objectives:**

Tracheostomy for coronavirus disease 2019 pneumonitis patients requiring prolonged invasive mechanical ventilation remains a matter of debate. This study analysed the timing and outcomes of percutaneous tracheostomy, and reports our experience of a dedicated ENT–anaesthetics department led tracheostomy team.

**Method:**

A prospective single-centre observational study was conducted of patients undergoing tracheostomy, who had been diagnosed with coronavirus disease 2019 pneumonitis, between 21st March and 20th May 2020.

**Results:**

Eighty-one patients underwent tracheostomy after a median (interquartile range) of 16 (13–20) days of invasive mechanical ventilation. Median follow-up duration was 32 (23–40) days. Of patients, 86.7 per cent were successfully liberated from invasive mechanical ventilation in a median (interquartile range) of 12 (7–16) days. Moreover, 68.7 per cent were subsequently discharged from hospital. On univariate analysis, there was no difference in outcomes between early (before day 14) and late (day 14 or later) tracheostomy. The mortality rate was 8.6 per cent and no deaths were tracheostomy related.

**Conclusion:**

Outcomes appear favourable when patients are carefully selected. Percutaneous tracheostomy performed via a multidisciplinary approach, with appropriate training, was safe and optimised healthcare resource utilisation.

## Introduction

Tracheostomy for weaning critically ill patients with coronavirus disease 2019 (Covid-19) who are receiving invasive mechanical ventilation remains a matter of debate. Controversy exists regarding timing, outcomes, prognosis, techniques to reduce aerosol generation and risk of transmission to healthcare workers. The mortality rate for those critically ill with Covid-19 pneumonitis is higher than for non-coronavirus disease viral pneumonia (50.7 per cent *vs* 22.0 per cent), as described in the Intensive Care National Audit and Research Centre (‘ICNARC’) report.^1^ For those patients who survive, the median duration of requirement for ventilation is reported at between 20 and 27 days.^1,2^

It is likely that a significant number of these patients may benefit from tracheostomy, as it has a recognised role in non-coronavirus disease populations to facilitate ventilatory weaning. These include reductions in: incidence of ventilator-associated pneumonia, duration of sedation, duration of mechanical ventilation and length of stay in critical care.^3,4^ Timing of tracheostomy insertion remains controversial, with no survival benefit demonstrated with earlier tracheostomy insertion.^5^

In the context of Covid-19, ENT-UK currently recommends performing tracheostomy on or after 14 days of endotracheal intubation.^6^ Additionally, the British Laryngological Association advises deferring tracheostomy until the patient has a positive end-expiratory pressure (PEEP) requirement of 10 cmH_2_O or less and a fraction of inspired oxygen of 0.4 or less.^7^ More recently, an international consensus guideline has recommended waiting at least 10 days, and only considering tracheostomy when the patient is showing signs of clinical improvement.^8^ Furthermore, a high incidence of thromboembolism has been reported in critically ill Covid-19 patients,^9^ necessitating therapeutic anticoagulation. These factors collectively pose significant peri-operative risks of hypoxia, de-recruitment and bleeding.

There has been understandable concern regarding the risk to healthcare workers when performing tracheostomy, which is mainly based upon evidence from the severe acute respiratory syndrome coronavirus 1 (SARS-CoV-1) pandemic.^10^ Current published evidence does not suggest a difference between surgical tracheostomy and percutaneous tracheostomy with regard to aerosol generation or outcomes,^11–13^ and whilst most have recognised this equipoise, some experts have published recommendations favouring a percutaneous approach.^14,15^

Guy's and St Thomas’ NHS Foundation Trust was one of the first centres in the UK to treat Covid-19 patients, and hence make decisions about tracheostomy. In March 2020, because of an anticipated surge in demand, we developed a dedicated ENT–anaesthetics department led tracheostomy team, through close collaboration with intensive care. Indications for tracheostomy were agreed, and intensive training and simulation was undertaken. The purpose was to rapidly up-skill our ENT clinicians to safely perform percutaneous procedures with appropriate modifications to minimise aerosol generation. Our initial protocol and recommendation are described in detail in our recent publication.^16^

This paper primarily aimed to analyse the intra-operative and post-tracheostomy outcomes in patients with Covid-19 pneumonitis requiring prolonged mechanical ventilation. In addition, we sought to analyse any effect of timing of tracheostomy upon outcomes in our cohort, and report our experience of both staff and patient safety.

## Materials and methods

### Study population and setting

This was a prospective observational cohort study of patients undergoing elective tracheostomy between 21st March and 20th May 2020 at Guy's and St Thomas’ Hospital. All patients included were diagnosed with laboratory confirmed Covid-19; all were critically ill with acute hypoxemic respiratory failure and receiving invasive mechanical ventilation. The decision to perform tracheostomy for anticipated prolonged respiratory weaning was made jointly by two critical care consultants after evaluation of each patient's clinical course and prognosis, considering the factors defined in our local guideline.^16^

By default, procedures were performed percutaneously at the bedside on the intensive care unit. In cases where there were potential contraindications to this approach (e.g. anatomical issues, a difficult upper airway, coagulopathy), a joint ENT–anaesthetics assessment was undertaken, including ultrasound of the neck, to determine the safest surgical approach and location (in which to undertake the tracheostomy) prior to proceeding.

**Fig. 1.**
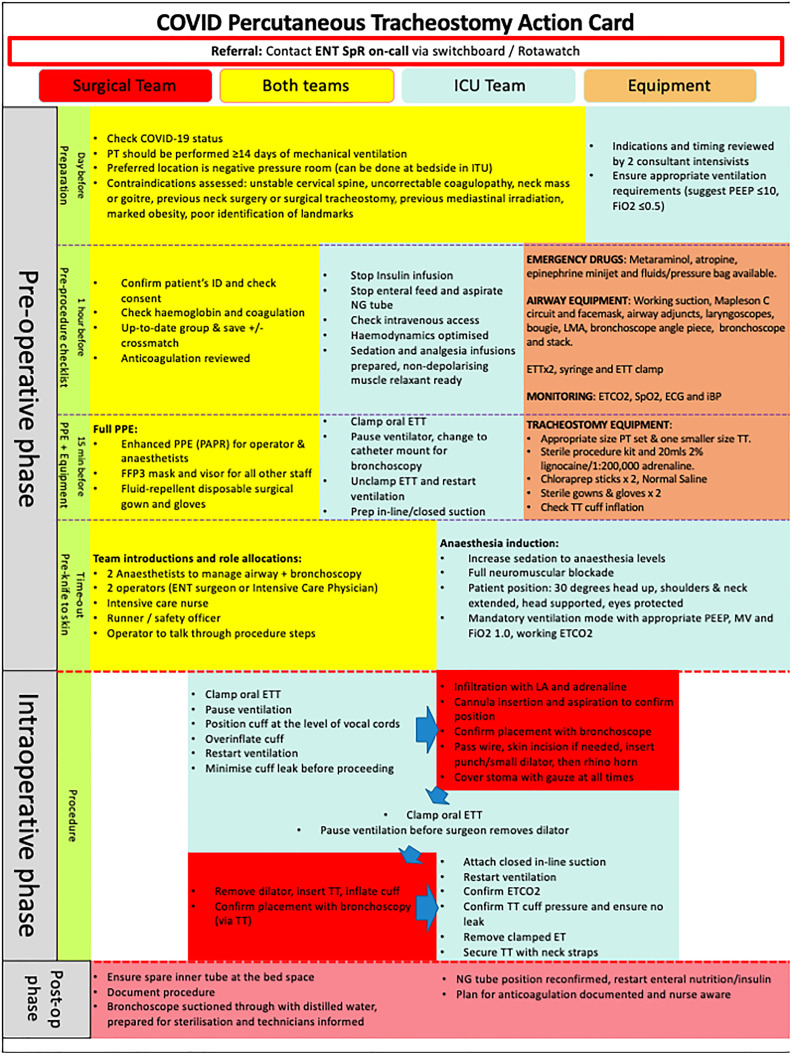
Percutaneous tracheostomy action card. Covid-19 = coronavirus disease 2019; SpR = specialist registrar; ICU = intensive care unit; PT = percutaneous tracheostomy; ITU = intensive therapy unit; PEEP = positive end-expiratory pressure; FiO_2_ = fraction of inspired oxygen; ID = identification; NG = nasogastric; LMA = laryngeal mask airway; ETT = endotracheal tube; ETCO_2_ = end-tidal carbon dioxide; SpO_2_ = oxygen saturation; ECG = electrocardiography; iBP = invasive blood pressure; min = minutes; PPE = personal protective equipment; PAPR = powered air-purifying respirator; FFP3 = filtering facepiece code 3; prep = prepare; TT = tracheostomy tube; MV = mechanical ventilation; LA = local anaesthetic; post-op = post-operative

The intensive care unit or dedicated ENT–anaesthetics tracheostomy team performed all procedures using the Tracoe® ‘percutan experc’ system according to a pre-agreed protocol (Figure 1). Patients were followed up until discharge from hospital or death.

This study was registered and approved with the institutional clinical governance department, and was approved as a service evaluation.

### Study objectives and measurements

Electronic medical records were used to obtain patients’ baseline characteristics, including age, gender, ethnicity, body mass index (BMI), number of very severe co-morbidities, Acute Physiology and Chronic Health Evaluation II (‘APACHE II’) score, and lowest recorded partial pressure of oxygen: fraction of inspired oxygen ratio within 24 hours of intensive care unit admission. We compared our cohort to the Intensive Care National Audit and Research Centre cohort that underwent advanced respiratory support during their critical care admission.^1^

With respect to tracheostomy, we analysed procedure timing (days since endotracheal intubation) and levels of respiratory support on the morning of the procedure. The recorded parameters were: PEEP, fraction of inspired oxygen, partial pressure of oxygen: fraction of inspired oxygen ratio, and active treatment with extracorporeal membrane oxygenation. Positive end-expiratory pressure and fraction of inspired oxygen were measured against our local guideline of PEEP of 10 cmH_2_O or less and fraction of inspired oxygen of 0.5 or less. Measurements of PEEP, fraction of inspired oxygen, and partial pressure of oxygen: fraction of inspired oxygen ratio for patients receiving extracorporeal membrane oxygenation were censored from analysis, as these would not accurately represent respiratory function in this cohort.

The laboratory serum biomarker C-reactive protein (CRP) was recorded on the day of the procedure, given its potential prognostic value in Covid-19 cases.^17,18^ In addition, we collected factors related to multi-organ dysfunction, including the requirement for vasopressors and/or renal replacement therapy.

Data on surgical technique, location, anticoagulant status, and intra- and post-operative complications were collected. The personal protective equipment worn by team members was recorded. All team members involved were surveyed and asked to report Covid-19 symptoms. These results were included if symptoms occurred within 5–14 days after a procedure.

The primary outcome was duration of ventilation post-tracheostomy. The endpoint of ventilation was defined as the day when all mechanical ventilation (including bilevel positive airway pressure and continuous positive airway pressure) was stopped for at least 24 hours.

Outcomes were also recorded from the day of tracheostomy until the day when: sedation was stopped (i.e. when intravenous sedative infusions of propofol, fentanyl, alfentanil or midazolam were stopped for at least 24 hours); the patient was discharged from the intensive care unit; decannulation of tracheostomy occurred; the patient was discharged from hospital; or death occurred. Where outcomes data were unobtainable, patients were censored from outcomes analysis.

### Statistical analysis

For univariate analysis of the effect of timing of tracheostomy, patients were divided into two groups according to current guidelines: early tracheostomy group – less than 14 days after endotracheal intubation; and late tracheostomy group – 14 days or more after endotracheal intubation.^6,7^

Baseline characteristics, respiratory support and inflammatory markers on the day of tracheostomy, and outcomes, were compared between early and late tracheostomy groups. A Kolmogorov test showed abnormal distribution, hence non-parametric tests were used. *P*-values of less than 0.05 were considered significant; these were calculated using the chi-square test for categorical variables and the Mann–Whitney U test for continuous variables. Spearman's test was used for correlation co-efficients. Statistical analysis was performed with SPSS version 20.0 software (IBM, Armonk, New York, USA).

## Results

### Baseline characteristics

A total of 263 patients were admitted to the intensive care unit for invasive mechanical ventilation between 3rd March and 5th May 2020; all had been diagnosed with laboratory confirmed Covid-19 and were critically ill with acute hypoxemic respiratory failure. Of the patients, 81 (30.8 per cent) underwent tracheostomy for prolonged respiratory weaning between 21st March and 20th May 2020.

The median (interquartile range) age of the 81 patients was 55 (46–61) years, with a male to female ratio of 2:1. Baseline demographics, which were compared to the Intensive Care National Audit and Research Centre cohort, are presented in Table 1.

**Table 1.** Baseline characteristics

	ICNARC cohort (*n* = 6395)*	Current study cohort			Univariate analysis (*p*-values)
Characteristics		Overall (*n* = 81)	Early tracheostomy (*n* = 24)	Late tracheostomy (*n* = 57)	
Age at admission (years)					
– Mean (SD)	58.8 (12.0)	52.9 (12.2)	58.4 (11.8)	50.6 (11.8)	
– Median (IQR)	60 (52–67)	55 (46–61)	60 (54–64)	52 (45–59)	NS
Sex (*n* (%))					
– Female	1767 (27.6)	26 (32.1)	9 (37.5)	17 (29.8)	NS
– Male	4626 (72.4)	55 (67.9)	15 (62.5)	40 (70.1)	NS
Ethnicity (*n* (%))					
– White	3770 (64.7)	35 (43.2)	11 (45.8)	24 (42.1)	NS
– Mixed	102 (1.8)	4 (4.9)	0 (0.0)	4 (7.0)	NS
– Asian	920 (15.8)	16 (19.8)	5 (20.8)	11 (19.3)	NS
– Black	624 (10.7)	23 (28.4)	7 (29.2)	16 (28.1)	NS
– Other	412 (7.1)	3 (3.7)	1 (4.2)	2 (3.5)	NS
BMI (kg/m^2^)					
– Mean (SD)	NA	30.9 (6.9)	30.0 (6.6)	31.2 (7.0)	
– Median (IQR)	NA	29.6 (25.7–34.6)	29.7 (23.8–34.4)	29.6 (26.1–34.6)	NS
BMI categories (*n* (%); kg/m^2^)					
– <18.5	32 (0.5)	0 (0)	0 (0)	0 (0)	NS
– 18.5 to ≤25	1468 (24.1)	18 (22.2)	8 (33.3)	10 (17.5)	NS
– 25 to ≤30	2165 (35.6)	24 (29.6)	4 (16.7)	20 (35.1)	NS
– 30 to ≤40	1970 (32.4)	32 (39.5)	11 (45.8)	21 (36.8)	NS
– >40	447 (7.3)	7 (8.6)	1 (4.2)	6 (10.5)	NS
Very severe co-morbidities					
– ≥1 co-morbidities (*n* (%))	NA	11 (13.6)	5 (20.8)	6 (10.5)	NS
– Mean (SD)	NA	0.2 (0.4)	0.3 (0.5)	0.1 (0.4)	
– Median (IQR)	NA	0 (0–0)	0 (0–0)	0 (0–0)	NS
APACHE II score					
– Mean (SD)	15.3 (5.1)	13.9 (3.7)	14.9 (3.4)	13.5 (3.8)	
– Median (IQR)	15 (12–18)	14 (11–17)	14.5 (13–17)	13 (11–17)	NS
PaO_2_:FiO_2_ ratio^†^					
– Mean (SD)	NA	126.8 (55.2)	133.1 (67.0)	124.2 (50.0)	
– Median (IQR)	114 (81.8–159.0)	119.1 (88.8–161.3)	121.5 (72.7–179.7)	119.1 (91.6–157.5)	NS

*Data (patient characteristics of those receiving advanced respiratory support) taken from Intensive Care National Audit and Research Centre report 12 June 2020.^1^ ^†^Derived from the lowest arterial blood gas measurement during the first 24 hours of care. ICNARC = Intensive Care National Audit and Research Centre; SD = standard deviation; IQR = interquartile range; NS = non-significant; BMI = body mass index; NA = not applicable; APACHE II = Acute Physiology and Chronic Health Evaluation II; PaO_2_:FiO_2_ = partial pressure of oxygen: fraction of inspired oxygen

### Timing of tracheostomy

The median (interquartile range) duration of invasive mechanical ventilation prior to tracheostomy was 16 (13–20) days. Fifty-seven tracheostomies (70.4 per cent) were performed on or after day 14. The (median (interquartile range)) levels of respiratory support required on the day of tracheostomy were as follows: PEEP of 8 (6–10) cmH_2_O, fraction of inspired oxygen of 0.35 (0.3–0.4), and partial pressure of oxygen: fraction of inspired oxygen ratio of 204.9 (171.2–249.0). Sixty-two patients (93.9 per cent) required PEEP of 10 cmH_2_O or less and fraction of inspired oxygen of 0.5 or less. The highest PEEP and fraction of inspired oxygen requirements were 14 cmH_2_O and 0.55 respectively.

The median (interquartile range) CRP was 116 (50–192) mg/l. Nineteen patients (23.5 per cent) were receiving vasopressors, 29 (23.5 per cent) were receiving renal replacement therapy and 15 (18.5 per cent) were on extracorporeal membrane oxygenation. The results are summarised in Table 2.

**Table 2.** Timing, respiratory parameters, CRP and receipt of organ support on day of tracheostomy

Parameter	Overall (*n* = 81)	Early tracheostomy (*n* = 24)	Late tracheostomy (*n* = 57)	Univariate analysis (*p*-values)
Day of tracheostomy				
– Mean (SD)	16.3 (4.6)	11.5 (1.8)	19.9 (5.0)	
– Median (IQR)	16 (13–20)	12 (11–12)	19 (16–22)	
CRP on day of tracheostomy (mg/l)				
– Mean (SD)	139.5 (102.6)	142.5 (106.6)	136 (100.8)	
– Median (IQR)	116 (50–192)	103 (77–197)	122 (50–185)	NS
Receipt of organ support on day of tracheostomy (*n* (%))				
– Extracorporeal membrane oxygenation	15 (18.5)	7 (29.2)	8 (14.0)	NS
– Renal replacement therapy	29 (35.8)	4 (16.7)	25 (43.8)	0.009
– Vasopressors	19 (23.5)	5 (20.8)	14 (24.6)	NS
– >1 organ support	12 (14.8)	3 (12.5)	9 (15.8)	NS
PEEP on day of tracheostomy (cmH_2_O)*	Overall (*n* = 66)	Early tracheostomy (*n* = 17)	Late tracheostomy (*n* = 49)	
– Mean (SD)	7.8 (2.0)	7.5 (1.6)	8.0 (1.6)	
– Median (IQR)	8 (6–10)	8 (6–8)	8 (6–10)	NS
– PEEP ≤10 (*n* (%))	74 (91.4)	17 (100)	45 (91.8)	NS
FiO_2_ on day of tracheostomy*				
– Mean (SD)	0.4 (0.1)	0.4 (0.1)	0.4 (0.1)	
– Median (IQR)	0.35 (0.3–0.4)	0.4 (0.3–0.45)	0.35 (0.3–0.4)	NS
– FiO_2_ ≤0.5 (*n* (%))	62 (93.9)	16 (94.1)	46 (93.9)	NS
PaO_2_:FiO_2_ ratio on day of tracheostomy*				
– Mean (SD)	215.7 (64.0)	197.1 (61.0)	222.2 (64.3)	
– Median (IQR)	204.9 (171.2–249.0)	217.0 (140.6–242.8)	202.8 (173.8–250.7)	NS

*Patients receiving extracorporeal membrane oxygenation on day of tracheostomy (*n* = 15) were excluded from analysis. CRP = C-reactive protein; SD = standard deviation; IQR = interquartile range; NS = non-significant; PEEP = positive end-expiratory pressure; FiO_2_ = fraction of inspired oxygen; PaO_2_:FiO_2_ = partial pressure of oxygen: fraction of inspired oxygen

### Procedures, complications and staff safety

Seventy-six procedures (93.8 per cent) were performed as percutaneous tracheostomy, and the remainder via a hybrid or open technique. A total of 78 procedures (96.3 per cent) were performed at the bedside on the intensive care unit; this included all percutaneous procedures, and 2 hybrid procedures.

All of the procedures were successfully performed as per the pre-agreed technique and location. Three patients (3.7 per cent) required planned transfer to the operating theatre (one hybrid and two open procedures), two of which were because of patient morbid obesity (BMIs of 43 kg/m^2^ and 61 kg/m^2^) with associated known difficult upper airways. The third patient was identified as having level VI calcific lymphadenopathy on computed tomography imaging; a level VI selective neck dissection was performed for both access and diagnostic purposes.

Overall, seven intra-operative complications (8.6 per cent) were recorded (Table 3). There was one episode (1.2 per cent) of intra-operative oxygen desaturations of less than 90 per cent on continuous monitoring. Post-operative complications are detailed in Table 3.

**Table 3.** Technique, and intra- and post-operative complications*

Parameter	Value (*n* (%))
Surgical technique	
– Percutaneous	76 (93.8)
– Open	2 (2.5)
– Hybrid	3 (3.7)
Intra-operative complications	
– Overall	7 (8.6)
– Misplacement	2 (2.4)
– Bleeding^†^	2 (2.4)
– Tracheal injury	1 (1.2)
– ETT cuff puncture	1 (1.2)
– Oxygen desaturations	1 (1.2)
Post-operative complications	
– Overall	7 (8.6)
– Bleeding^‡^	5 (6.2)
– Suprastomal granulations	2 (2.4)

* Total *n* = 81. ^†^Both patients were on prophylactic low molecular weight heparin that was not omitted pre- or post-procedure; bleeding was managed with local direct pressure only. ^‡^Three of the five patients (60 per cent) were on continuous unfractionated heparin infusions at the time of bleeding, and two of the five (40 per cent) were on prophylactic dose low molecular weight heparin; anticoagulant therapy was omitted on the day of post-operative bleeding. ETT = endotracheal tube

Overall, 25 patients (30.9 per cent) were receiving therapeutic and 50 (61.7 per cent) were receiving prophylactic anticoagulants around the time of tracheostomy. Bleeding (in 7.6 per cent) was documented as either oozing from the stoma or as the suctioning of fresh blood via tube. Three (60 per cent) of these patients were on continuous unfractionated heparin at the time of bleeding, with the remainder on prophylactic low molecular weight heparin. All cases were managed with suctioning, intravenous tranexamic acid and/or local injection of lidocaine with adrenaline. No patients required blood transfusion or operative intervention. One patient (1.2 per cent) required microlaryngoscopy and debridement of suprastomal granulations to facilitate decannulation.

All operators and assistants wore powered air-purifying respirators or filtering facepiece code 3 (FFP3) masks and shield visors, along with a double-layer, fluid-repellent, disposable surgical gown and gloves. A team of five healthcare workers were present for every procedure, including two airway operators, two surgeons, and a runner/safety officer. There were 71 healthcare workers (average of 5.7 procedures each) involved in the tracheostomy teams. Of those surveyed, none developed Covid-19 symptoms within the relevant 5–14-day post-exposure window. The survey response rate was 81.7 per cent. Four healthcare workers reported having Covid-19 prior to undertaking the procedures.

### Outcomes and mortality

The median (interquartile range) follow-up time was 32 (23–40) days post-tracheostomy insertion. Sixty-five patients (86.7 per cent) had been liberated from invasive mechanical ventilation, with a median (interquartile range) post-tracheostomy duration of ventilation of 12 (7–16) days. Moreover, 59 (86.7 per cent) of patients had been successfully decannulated and 44 (68.7 per cent) had been successfully discharged from hospital in a median (interquartile range) duration of 19 (15–27) days and 32 (25–39) days, respectively.

**Fig. 2.**
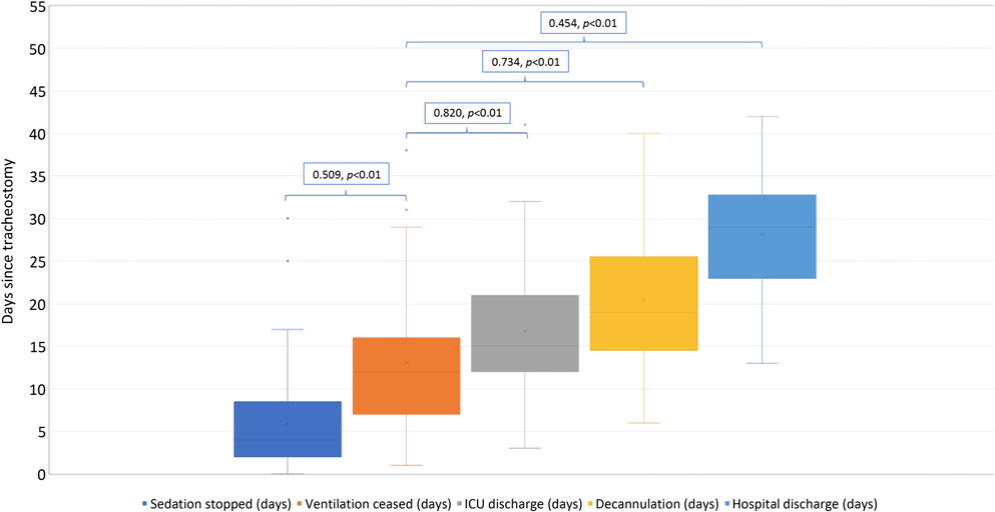
Boxplot showing outcomes after tracheostomy (median, mean, interquartile range and range). Spearman correlations between stopping ventilation and other outcomes are displayed. ICU = intensive care unit

Correlations between outcomes were analysed. We observed a strong correlation between stopping ventilation, decannulation (r = 0.734, *p* < 0.01) and hospital discharge (r = 0.454, *p* < 0.01). Detailed outcome data are presented in Table 4 and Figure 2. There were no significant differences between the early and late groups at baseline (Table 1), on the day of procedure (Table 2) or in post-tracheostomy outcomes (Table 4).

**Table 4.** Patient outcomes and mortality

Parameter	Overall (*n* = 81)	Early tracheostomy (*n* = 24)	Late tracheostomy (*n* = 57)	Univariate analysis (*p*-values)
Follow-up period				
– Mean (SD) (days)	32.6 (14.9)	36.4 (16.2)	31.1 (14.2)	
– Median (range) (days)	32 (23–40)	37 (24–42)	31 (22–37)	NS
Sedation was stopped*				
– Patients (*n* (%))	72 (90.0)	19 (82.6)	53 (93.0)	NS
– Mean (SD) (days)	6.2 (6.6)	5.6 (4.1)	6.4 (7.4)	
– Median (IQR) (days)	4 (2–9)	5 (3–7.5)	3.5 (2–9)	NS
Ventilation was stopped^†^				
– Patients (*n* (%))	65 (86.7)	18 (81.2)	47 (88.7)	NS
– Mean (SD) (days)	13.2 (8.3)	14.3 (7.1)	12.9 (8.7)	
– Median (IQR) (days)	12 (7–16)	13.5(8–21)	12 (7–16)	NS
ICU discharge^‡^				
– Patients (*n* (%))	62 (86.1)	18 (81.2)	44 (88.0)	NS
– Mean (SD) (days)	18.2 (10.1)	17.6 (8.2)	18.5 (10.8)	
– Median (IQR) (days)	16 (12–23)	15 (11–25)	17 (12–21)	NS
Decannulation**				
– Patients (*n* (%))	59 (83.1)	17 (80.9)	42 (84.0)	NS
– Mean (SD) (days)	21.4 (9.3)	22.3 (8.0)	21.0 (9.9)	
– Median (IQR) (days)	19 (15–27)	23 (15–27)	18 (15–24)	NS
Hospital discharge^§^				
– Patients (*n* (%))	44 (68.7)	14 (73.7)	30 (66.7)	NS
– Mean (SD) (days)	32.9 (11.5)	38.4 (14.8)	30.3 (8.8)	
– Median (IQR) (days)	32 (25–39)	37 (29–41)	31 (24–37)	NS
Status of survival (*n* (%))				
– Alive	74 (91.4)	21 (87.5)	53 (93.0)	NS
– Dead	7 (8.6)	3 (12.5)	4 (7.0)	NS

Missing data were a result of patients being transferred back to local units prior to outcomes being reached. *Data available for 80 of 81 patients (23 of 24 early and 57 of 57 late tracheostomy cases); ^†^data available for 75 of 81 patients (22 of 24 early and 53 of 57 late tracheostomy cases); ^‡^data available for 72 of 81 patients (22 of 24 early and 50 of 57 late tracheostomy cases); **data available for 71 of 81 patients (21 of 24 early and 50 of 57 late tracheostomy cases); and ^§^data available for 64 of 81 patients (19 of 24 early and 45 of 57 late tracheostomy cases). SD = standard deviation; NS = non-significant; IQR = interquartile range; ICU = intensive care unit

The overall mortality rate was 8.0 per cent (7 out of 81). Deaths occurred at a median (range) of 14 (5–40) days post-tracheostomy and 29 (17–50) days post-intubation. The cause of death was ventilator-dependant respiratory failure in all cases. There were no tracheostomy related deaths. For the subset receiving extracorporeal membrane oxygenation at the time of tracheostomy, the mortality rate was 26.7 per cent (4 out of 15); all of these patients were still receiving extracorporeal membrane oxygenation up until their time of death. The remaining cohort had a mortality rate of 4.5 per cent (3 out of 66).

## Discussion

Despite a huge effort to produce guidelines to support decision-making and procedure for tracheostomy in Covid-19 patients, there still remains a paucity of published data on outcomes, complications and safety.

Our population was younger, and with a higher proportion of Black, Asian and minority ethnic groups, compared to the Intensive Care National Audit and Research Centre dataset.^1^ Our diverse local population may account for some of this variation. This study includes some of the most critically ill Covid-19 patients, with a significant proportion requiring the support of two or more organ systems, and a very high proportion receiving extracorporeal membrane oxygenation on the day of tracheostomy.

In this study, 29.6 per cent of patients underwent tracheostomy before the current recommended minimum time of 14 days. There was good adherence to departmental^16^ and British Laryngological Association^7^ guidelines with regard to levels of respiratory support, irrespective of timing of tracheostomy. We compared those who underwent tracheostomy before and after day 14, and observed no difference in outcomes. Furthermore, there was no difference in the baseline characteristics, or markers of disease severity on admission (Acute Physiology and Chronic Health Evaluation II score, and partial pressure of oxygen: fraction of inspired oxygen ratio) or on the day of tracheostomy (PEEP measurement, partial pressure of oxygen: fraction of inspired oxygen ratio, and CRP level). It therefore appears that timing may not be the single factor that should determine the decision to proceed with tracheostomy, and the broader clinical picture should be considered. Specifically, levels of respiratory support and other trends in markers of disease severity may play a significant role, and this warrants further study.

Overall complication rates were comparable to rates in existing published literature for non-coronavirus disease tracheostomy cases.^5,19^ Furthermore, rates of post-operative bleeding appear lower than for open techniques in Covid-19 patients,^20^ despite a high proportion of our cohort receiving therapeutic anticoagulant therapy. Misplacement rates appeared higher than previously published (1.5 per cent),^5^ which may be a result of small study size, but could reflect early attempts to avoid bronchoscope usage in order to minimise aerosol generation;^15^ our protocols were adopted to mitigate these risks.

**Fig. 3.**
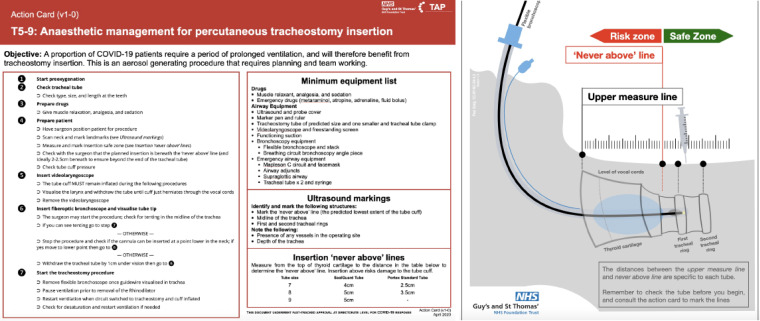
Anaesthetic action card. COVID-19 = coronavirus disease 2019

Cuff puncture also presented a particular concern because of the risk of aerosol generation and the potential for prolonged loss of ventilation. This highlighted an important point, as endotracheal tube cuffs used on intensive care unit are longer than those commonly used in the operating theatre setting. In response, our anaesthetic colleagues developed a specific action card (Figure 3), utilising real-time ultrasound to minimise risk.

Intra-operative desaturations were reported as the most common complication in a multicentre interim report.^20^ By following our protocol to minimise periods of apnoea, and deferring tracheostomy until levels of ventilatory support were favourable, we were able to mitigate this risk in our study. This may also reflect patients in our study being in the later stages of recovery from illness by the time tracheostomy was performed.

Whilst there is still not consensus on which technique generates less aerosol,^10,14,15^ our standards of personal protective equipment (including powered air-purifying respirators) currently appear safe, with no identified healthcare worker transmission. The response rate in our follow-up survey of symptoms in this study may limit the accuracy of our findings; however, its results are reflective of other reports of tracheostomy in Covid-19.^11,20,21^

The obvious value of our bedside team-based approach has been to optimise resource utilisation during hugely challenging times. Moreover, the need to transfer patients to the operating theatre has been safely avoided in most cases, as a result of careful planning. Furthermore, our unique approach of ENT and anaesthesia teams performing bedside procedures helped to relieve intensive care unit staff and exploit the capacity of our anaesthetists as the number of primary intubations started to fall and the consequent demand for tracheostomies increased. The modification of our process in response to early learning, and our multidisciplinary collaboration along with extensive training and simulation, has enabled us to safely deliver this service, in line with the approach recommended by the National Patient Safety Improvement Programme.^8^

Whilst there have been a number of reports on percutaneous tracheostomy in Covid-19 patients,^11–13,20,21^ these have variable data on associated post-tracheostomy outcomes, and are limited by short follow-up periods. The current study provides one of the most comprehensive reports on outcomes to date. We observed that most patients were successfully decannulated during our follow-up period. A limitation of the outcomes reported is that some data were unobtainable; this was because of patients being transferred back to local units prior to all outcome measures being recorded. Whilst we demonstrated correlation in outcomes measures, we cannot account for the levels of variation observed in the duration of weaning and rehabilitation; research into disease-specific factors that may influence the clinical course is urgently needed.

• The coronavirus disease 2019 (Covid-19) pandemic has placed unprecedented demand on critical care for invasive mechanical ventilation
• A significant number of patients may benefit from tracheostomy, but evidence on optimal timing, technique and efficacy is lacking
• In 81 patients, percutaneous tracheostomy complications were comparable to non-Covid-19 populations, and lower than for open tracheostomy in Covid-19 patients
• There were no reported episodes of healthcare worker transmission and no tracheostomy related deaths
• Of patients, 86.7 per cent were liberated from invasive mechanical ventilation in a median (interquartile range) of 12 (7–16) days
• An airway management algorithm for percutaneous tracheostomy utilising ultrasound and bronchoscopy is presented

The mortality rate in this study is lower than the 31.5 per cent 30-day mortality reported in a large trial of critically ill patients who underwent prolonged mechanical ventilation;^5^ however, a small number of patients in this cohort were still receiving mechanical ventilation at the time of reporting and so the final mortality rate may eventually be higher.

Mortality rates were higher amongst patients receiving extracorporeal membrane oxygenation at the time of tracheostomy, and this may call into question the value of performing tracheostomy on this subset of patients. In non-coronavirus disease populations receiving extracorporeal membrane oxygenation, consideration of tracheostomy is recommended for patients deemed to be on a trajectory to recovery, as it has the same perceived benefits of reduced sedation requirements and a lower incidence of ventilator-associated pneumonia.^22–24^ In this study, patients receiving extracorporeal membrane oxygenation were selected for tracheostomy according to the same principles as non-extracorporeal membrane oxygenation patients. This pragmatic approach was taken given the current lack of literature surrounding extracorporeal membrane oxygenation in Covid-19 patients. Early case series of Covid-19 patients have shown mortality rates in the region of 35.3–44 per cent for this group,^25,26^ but the specific role of tracheostomy is not yet fully understood.

A key limitation of this study is that it describes a single-centre experience only, with a relatively small sample size, thus limiting the generalisability of our findings. However, it does represent a prospective study where patients were treated in accordance with current national guidance, and where procedures were performed according to a defined protocol.

Given the current variation in practice and outcomes, there is an urgent need for multicentre analysis, to better understand the optimal timing, indications and outcomes. Although tracheostomy has well-documented benefits for non-coronavirus disease patients requiring prolonged respiratory weaning,^3,4^ it is not possible to draw any firm conclusions on the overall positive or negative impact for tracheostomy patients with Covid-19 in this or other published studies.^11,13,20,21,27,28^ Prospective, randomised clinical trials are required to further address this for further waves of the Covid-19 pandemic.
